# Characteristics of strong ground motions in the 2014 *M*_s_ 6.5 Ludian earthquake, Yunnan, China

**DOI:** 10.1007/s10950-015-9532-x

**Published:** 2015-10-24

**Authors:** J. J. Hu, Q. Zhang, Z. J. Jiang, L. L. Xie, B. F. Zhou

**Affiliations:** 1 Key Laboratory of Earthquake Engineering and Engineering Vibration, Institute of Engineering Mechanics, China Earthquake Administration, 29 Xuefu Road, Harbin, 150080 China; 2 Harbin Institute of Technology, Harbin, 150080 China

**Keywords:** Ludian earthquake, Ground motion, Attenuation relationship, Significant duration, Site amplification

## Abstract

The 2014 *M*_s_ 6.5 (*M*_w_6.1) Ludian earthquake occurred in the eastern Sichuan–Yunnan border region of western China. This earthquake caused much more severe engineering damage than the usual earthquakes with the same magnitude in China. The National Strong Motion Network obtained large set of ground motion recordings during the earthquake. To investigate the engineering interested characteristics of ground motion from Ludian earthquake and compare it with the *M*_w_ 7.9 Wenchuan and the *M*_w_ 6.6 Lushan earthquakes in western China, studies on the ground motion field, attenuation relationship, distance dependence of significant duration, and site amplification were carried out. Some conclusion is drawn. Specifically, the ground motion field reveals a directional feature, and the distribution characteristics of the two horizontal components are similar. The attenuation relationship for Ludian earthquake is basically consistent with the ground motion prediction equation (GMPE) for western China, except the slight smaller than the GMPE predicted at short periods. The distance dependences of ground motion duration are different in Sichuan and Yunnan regions due to the local physical dispersion and *Q* value. The site amplification factors are dominated by linear site response for lower reference ground motion, but the nonlinearity becomes notable for higher reference ground motion. This feature is basically consistent with the empirical model for western China. All the results indicate that the spatial distribution of ground motion, the attenuation characteristics, and the site amplification effect should be considered in characterization of near-field ground motion.

## Introduction

The *M*_s_ 6.5 (*M*_w_ 6.1) Ludian earthquake occurred at 16:30 on August 3, 2014 (UTC+08:00) in Yunnan province, China. The epicenter was located at latitude 27.189 °N, longitude 103.409 °E, with a focal depth of 12 km according to the US Geological Survey (USGS). As a result of high vulnerability of most of the structures in this region, the earthquake caused huge engineering damage. More than 600 fatalities and,3000 injuries had been reported as of a week after the earthquake. The seismic intensity from field survey in the magistoseismic area reached IX, and the affected area with the seismic intensity over VI is about 10,350 km^2^.

In recent years, there have been several destructive earthquakes (*M* > 6.0) occurred in western China, such as the Wenchuan *M*_s_ 8.0 (*M*_w_7.9) earthquake in 2008, the Lushan *M*_s_ 7.0 (*M*_w_ 6.6) earthquake in 2013, the Minxian–Zhangxian *M*_s_ 6.6 (*M*_w_ 6.0) earthquake in 2013, and this *M*_w_ 6.1 Ludian earthquake in 2014. Recordings from destructive earthquakes were significantly useful for study of strong ground motion characteristics and developing new ground motion prediction equation (GMPE) for this high seismic risk region.

Empirical ground motion attenuation relationships are mainly developed from real strong ground motion recordings. The Next Generation Attenuation (NGA) project developed a set of updated attenuation relationships, which greatly promoted the worldwide study of new ground motion attenuation relationships (Abrahamson et al. 2013; Boore et al. 2013; Campbell and Bozorgnia 2013; Chiou and Youngs 2013; Idriss 2013). In China, due to the lack of strong motion recordings before the great Wenchuan earthquake, the traditional ground motion attenuation relationships were mainly transformed from the seismic intensity attenuation relationships indirectly (Yu and Wang 2006). This transforming method is based on the assumption that there exists a correspondence between the ground motion attenuation and seismic intensity attenuation. Apparently, this approach lacks of physical and theoretical background and it cannot reflect the significance features of near-fault effect, site condition effect, and fault mechanics on ground motions. After the *M*_w_ 7.9 Wenchuan earthquake in 2008, some attempts have been made to build ground motion attenuation relationships from ground motion recordings of the Wenchuan main and aftershocks (Lu et al. 2010; Zhang et al. 2012; Hu et al. 2015).

However, the Wenchuan earthquake database was too limited for lacking of earthquake events for 6.0–7.0 magnitude (Zhang et al. 2013), thus the developed attenuation relationships are basically only applicable for small to moderate magnitude earthquakes. Consequently, their results can hardly be used in the near fault regions for large earthquakes which are more interested in engineering. Therefore, in this paper, we aimed at characterizing the ground motion features of the *M*_s_ 6.5 Ludian earthquake. Specifically, the distribution of ground motion, the attenuation of ground motion amplitude, the distance-dependence of ground motion duration, and the site amplification characteristics were studied. In addition, these characteristics are compared with those of the *M*_w_ 7.9 Wenchuan and *M*_w_ 6.6 Lushan earthquakes to study the differences and similarities of strong motion in western China.

## The 2014 *M*_s_ 6.5 Ludian earthquake

### Tectonic background and fault model

**Fig. 1:**
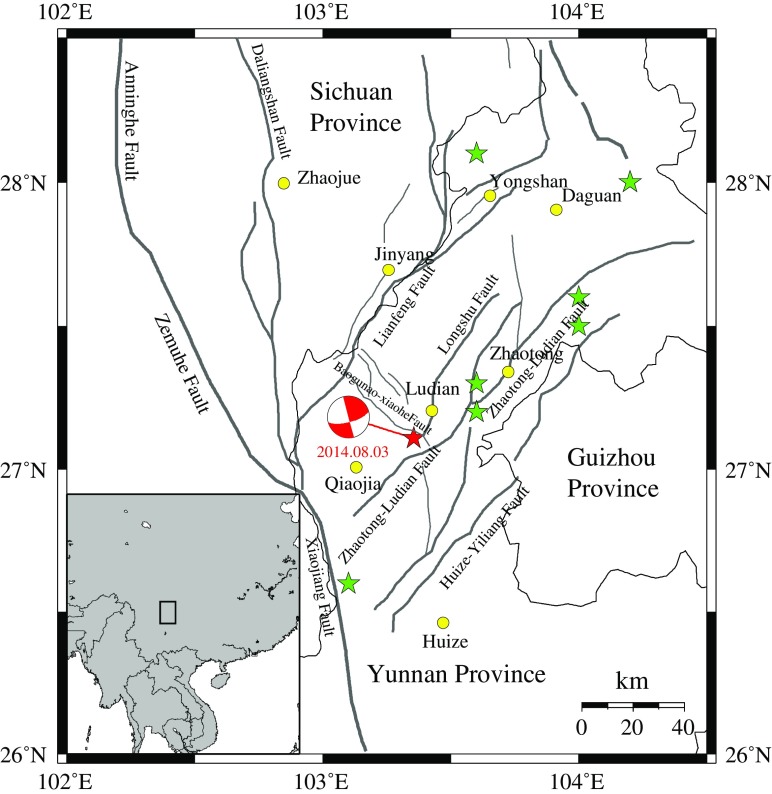
Tectonic background of the 2014 *M*_s_ 6.5 Ludian earthquake (adopted from Liu et al. 2014). The *red star* represents the epicenter of the 2014 *M*_s_ 6.5 Lushan earthquake; the *green stars* represent the other earthquakes

**Fig. 2:**
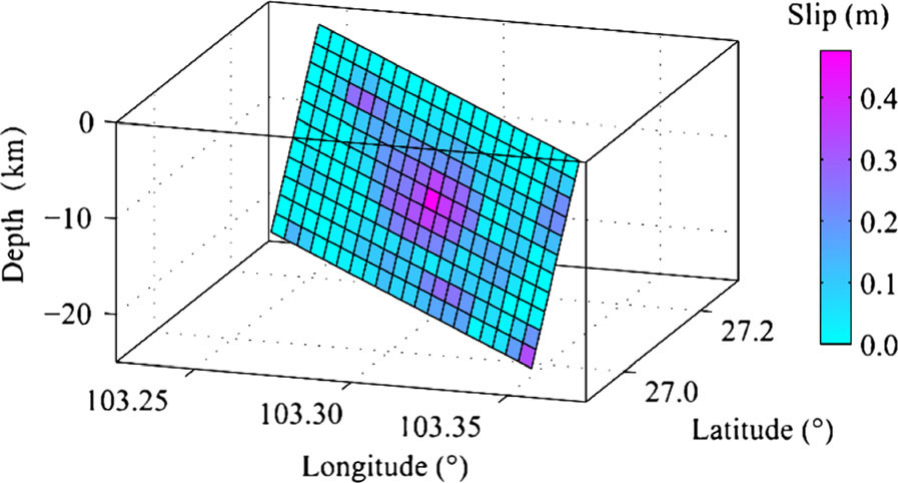
Finite fault model of the 2014 *M*_s_ 6.5 Ludian earthquake

The 2014 *M*_s_ 6.5 Ludian earthquake occurred in the Zhaotong–Ludian fault zone. This fault zone is composed of two NE directional faults located in the eastern Sichuan–Yunnan border region (see Fig. 1) (adopted from Wen et al. 2013). In the last decades, a series of earthquakes had occurred in this region. Such as the Ludian *M*_s_ 5.0 and *M*_s_ 5.1 earthquakes in 2003, the Ludian *M*_s_ 5.6 earthquake in 2004, the twice Yanjin *M*_s_ 5.1 earthquakes in 2006, the Yiliang *M*_s_ 5.6 and *M*_s_ 5.7 earthquakes in 2012, and the Yongjia *M*_s_ 5.3 earthquake in 2014. The frequently occurred earthquakes reflect the high seismicity in this tectonic region (Liu et al. 2014). Figure 1 shows the earthquakes for *M*_s_ > 5.0 in the last decades, where the solid line represents the active fault. For the 2014 *M*_s_ 6.5 Ludian earthquake, according to the source inversion results (Zhang et al. 2014; Liu et al. 2014), the rupture slips was mainly distributed in an area with about 15 km length and 10 km width on the fault plane, the azimuth strike angle is 162°, the dip angle is 86°, and the rake angle is 6°. Figure 2 shows the finite fault model by Zhang et al. (2014), all details of the fault parameters are shown in Table 1.

**Table 1:** Parameters of Lushan earthquake and geometry of fault plane

Moment magnitude	6.1	Strike (degree)	162
Rupture length (km)	16.3	Dip (degree)	86
Rupture width (km)	7.2	Rake (degree)	6
Hypocenter latitude	27.245 °N	Fault type	Strike slip
Hypocenter longitude	103.427 °E	Hypocenter location (strike direction)	0.5
Hypocenter depth (km)	10	Hypocenter location (dip direction)	0.75

### Engineering damage and typical ground motion

**Fig. 3:**
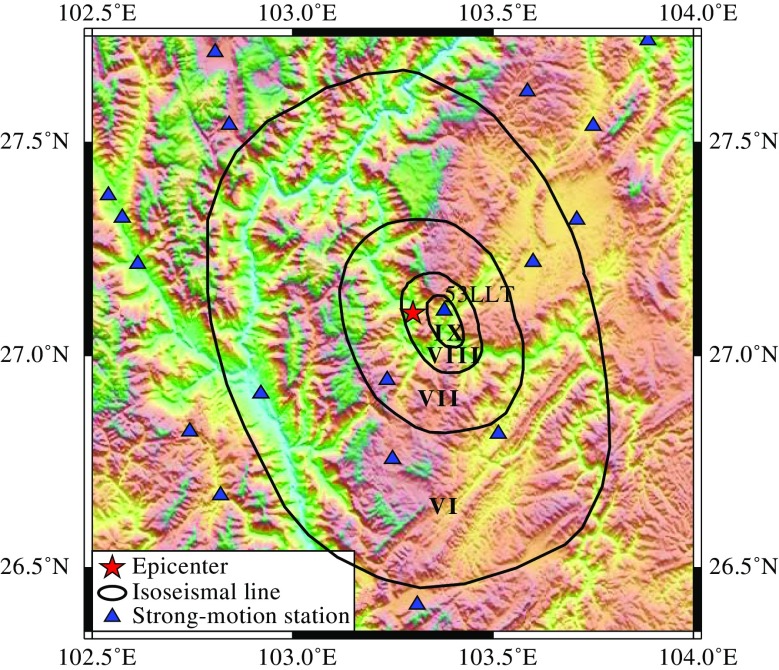
Intensity map and strong-motion stations (adopted from Ji et al. 2014)

The 2014 *M*_s_ 6.5 Ludian earthquake caused serious engineering damage and huge economic losses. It caused a death toll of 617 with 112 people missing. The engineering structures in the epicenter areas include masonry structures, reinforced concrete (RC) structures, wood structures, and adobe structures. Figure 3 shows the intensity map of the 2014 Ludian earthquake derived from field survey, strong ground motion data, and remote sensing image by the China Earthquake Administration (Ji et al. 2014). The intensity contour map shows a distinct NNE distribution of engineering damage along the fault strike.

**Fig. 4:**
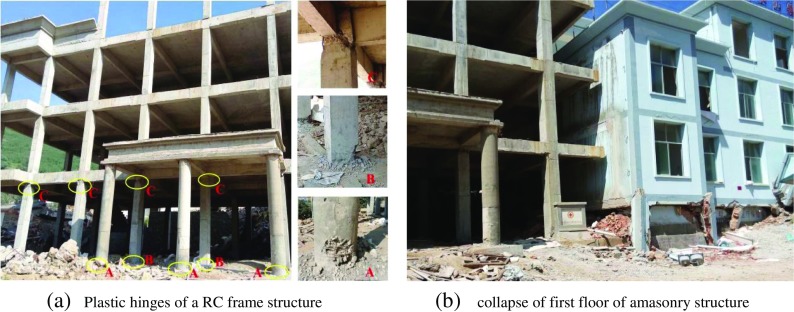
Engineering damage of structures in Loutoushan town close to the epicenter. **a** Plastic hinges of a RC frame structure **b** Collapse of first floor of a masonry structure

**Fig. 5:**
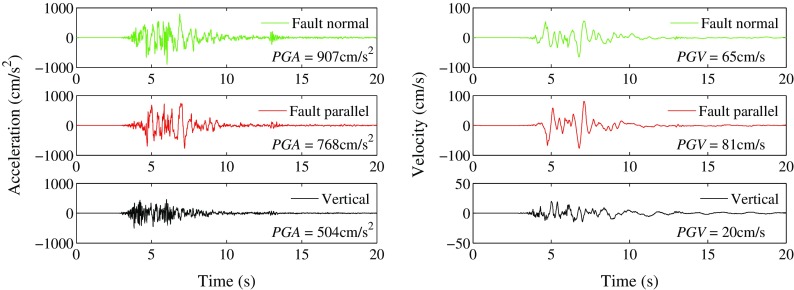
Acceleration and velocity time history in Loutoushan strong motion station (53LLT)

**Fig. 6:**
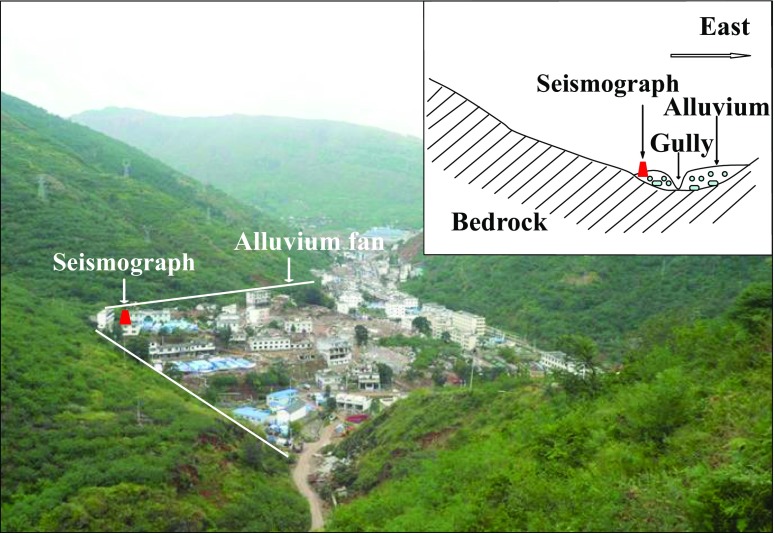
Topography and profile of Loutoushan strong motion station (53LLT) (provided by Cui Jianwen)

Except the destroys and collapses of many rural constructions with no consideration of seismic design, some new built RC structures and masonry structures were also destroyed in the seismic intensity IX areas. As a typical example, Fig. 4 shows the damage of a RC frame structure under construction and the collapse of an adjacent masonry structures in Loutoushan town close to the epicenter. In Fig. 4a, lots of plastic hinges appeared at the top and the foot of the frame columns in the first floor, the expected beam hinge failure mechanism did not show up (Lu et al. 2014; Zhou and Zhang 2014). In Fig. 4b, as a contrast, the first floor of an adjacent masonry building was completely collapsed. Figure 4 shows the acceleration and velocity time histories of the Loutoushan strong motion station (53LLT) which is about 0.2 km to the destroyed building in Fig. 4. The fault-normal (FN), fault-parallel (FP), and vertical (UD) components of the 53LLT station are given in Fig. 5. The site condition is complicated for this station, Fig. 6 (provided by Cui Jianwen) shows the topography and profile, but there is no detailed borehole profile or shear wave velocity data for this station. It can be seen from Fig. 6 the station located in a valley and on the alluvium which could induce the amplification effect.

## Strong ground motion database

**Fig. 7:**
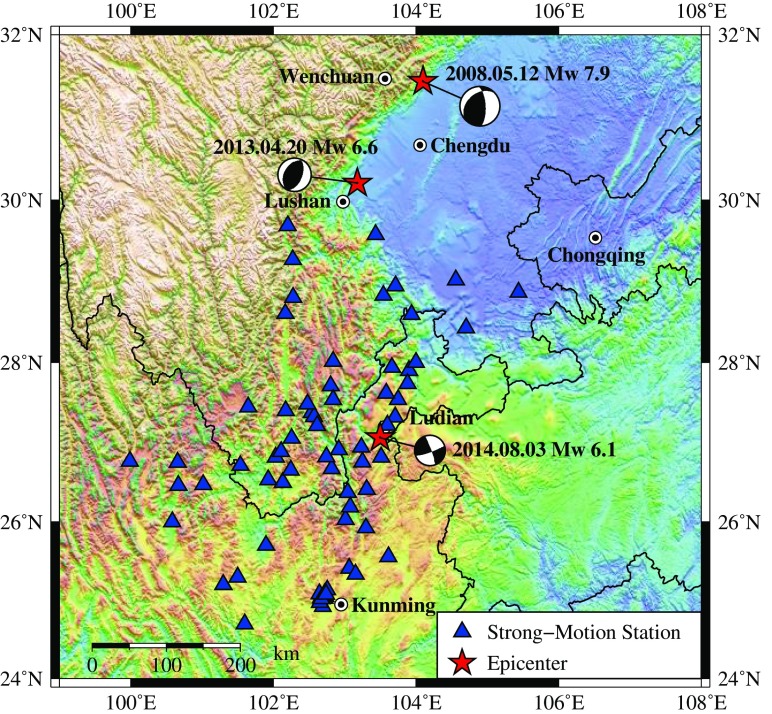
Strong motion stations during the 3 August 2014 *M*_s_ 6.5 Ludian earthquake and epicenters of Ludian, Lushan, and Wenchuan earthquakes

During the main shock, a total of 74 strong motion seismometers owning to the China Digital Strong Motion Observation Network (http://www.csmnc.net/) were released. The strong motion stations and epicenters of Ludian, Lushan, and Wenchuan earthquakes are shown in Fig. 7. Among the 74 seismometer stations, there are five structural array stations, four cave stations, five rock site stations, and 60 soil site stations. Table 2 shows the number and percentage of stations in different distance ranges for the free field stations (rock and soil). For all the original recorded ground motions, the largest peak ground acceleration (PGA) appeared in the East–West component of Longtoushan station (LLT) in Ludian County, with a rupture distance of 9.4 km to the fault. The rotated maximum PGA appeared in the fault normal component (see Fig. 5).

**Table 2:** Distribution of free field stations in different distance ranges

Rupture distance (km)	Number of stations	Percentage
0–40	5	8
40–100	17	26
100–200	19	29
>200	24	37

After baseline correction and band-pass filtering to the raw data, strong motion recordings in Ludian earthquake were selected for this study by using the following criteria: (1) recordings are representative of free-field; (2) the rupture distance is not greater than 200 km; (3) both horizontal components are available and the PGA is greater than 5 cm/s^2^; and (4) both horizontal components are with usable periods from 0.1 s to 5 s. So the final dataset for attenuation relationship studies consists of recordings from 26 stations, listed in Table 3.

**Table 3:** Dataset of records for attenuation relationship studies

No.	Station ID	*R*_rup_ (km)	Site	HP (Hz)	LP (Hz)
01	53LLT	9.4	Soil	0.08	30
02	53LDC	18.2	Rock	0.08	30
03	53ZTT	26.2	Rock	0.08	30
04	53QQC	30.9	Soil	0.08	30
05	53YML	36.8	Soil	0.08	30
06	53ZJA	39.2	Soil	0.10	20
07	53HYC	42.1	Soil	0.08	30
08	53QJX	49.1	Soil	0.08	30
09	53QJT	55.8	Rock	0.08	15
10	53BTT	65.1	Soil	0.08	20
11	53HZH	72.7	Soil	0.08	20
12	53YSZ	73.0	Soil	0.10	20
13	51HDX	76.0	Soil	0.08	15
14	51NNS	77.8	Soil	0.10	15
15	51HDQ	80.3	Soil	0.08	15
16	51PGB	83.1	Soil	0.08	15
17	51HZX	85.5	Soil	0.10	20
18	51PGD	87.7	Soil	0.20	15
19	53DJL	94.1	Soil	0.20	15
20	53DTB	96.4	Rock	0.10	20
21	51PGQ	96.6	Soil	0.10	20
22	51HLY	113.6	Soil	0.10	20
23	53DTD	114.1	Rock	0.08	15
24	51HLD	128.3	Soil	0.10	15
25	53DFZ	133.3	Soil	0.20	10
26	51MYS	141.2	Soil	0.10	15

*HP*, *LP*, high pass and low pass cutoff frequency of filter, respectively

In this study, the closest distances from stations to the fault (*R*_rup_) were used as rupture distance metric. To obtain the rupture distance for each station, instead of using the finite fault model, an estimation method by Chiou and Youngs (2008) was adopted. This is because the finite fault model usually presents an extraordinary large area including all the details of slip produced by the rupture. In that case, it could underestimate the rupture distance, especially in near fault area. Chiou and Youngs (2008) presented a distance estimation method for ground motion station without finite rupture model. By using this method, the empirical relationship of rupture size and magnitude by Wells and Coppersmith (1994) is adopted to estimate the rupture length and width. The location of hypocenter in the seismic rupture is specified at the middle of rupture along the strike direction and a quarter of width from bottom of rupture along the dip direction. All details about seismic rupture used in this study are shown in Table 1.

In addition, because the near-fault effects may have different influences on different components of ground motion, the original horizontal North–South and East–West directional components were rotated into fault-normal (FN) and fault-parallel (FP) components according to the fault geometry.

## Characteristics of strong ground motions

### Ground motion field

**Fig. 8:**
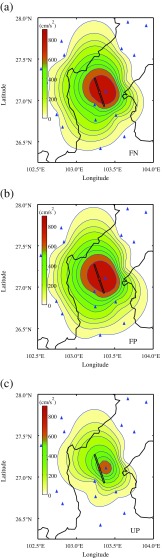
Ground motion field of response spectra (*T* = 0.3 s) FN component (**a**), FP component (**b**), Vertical component (**c**). The *red star* represents the epicenter of the 2014 *M*_s_ 6.5 Lushan earthquake, the *triangles* represent the strong-motion stations, and the *thick line* is the border between provinces

Taking advantage of the acceleration recordings in the 2014 Ludian earthquake, the contour maps of PGA and spectral acceleration (*S*_a_) for different periods were analyzed, aiming at better understanding of the distribution characteristics of ground motions. Figure 8 shows the ground motion fields of response spectra (*T* = 0.3 s). Where part (a), (b), and (c) represents the FN, FP, and UP (vertical) component, respectively. The spectral period of 0.3 s was selected because the local engineering structures were mostly low rise masonry buildings, and their natural periods are about 0.3 s. The contour maps indicated a directional distribution of spectral acceleration along the fault strike direction due to the strike slip mechanism; this feature is consistent with the intensity contour map in Fig. 3. To view the spectral amplitude characteristics of the two orthogonal components, seeing from Fig. 8 part (a) and (b), it is very clear that there is almost no difference in the attenuation and distribution characteristics of the FN and FP components, but apparently the amplitude of the vertical component is smaller than the horizontal ones.

From Fig. 8, it can be seen that the ground motion are higher around the 53LLT station, this may attribute to the topography effect or the site effect of 53LLT station (see Fig. 6). Seeing from Fig. 6 (provided by Cui Jianwen), the 53LLT station located on the alluvium fan, in front of the hill, so there is the possibility of amplification effect by topography or the soil. But unfortunately, there is no borehole profile for this station to carry out a detailed analysis of the site effect (Cui et al. 2014; Ji et al. 2014).

### Attenuation of ground motion

Geometric mean values of the two horizontal components were used to evaluate the attenuation characteristic of ground motion amplitude of the 2014 *M*_s_ 6.5 Ludian earthquake. Various functional forms of attenuation relationships had been tried to regress the model parameters, but the data could hardly determine the geometrical-spreading factor and the anelastic attenuation coefficient simultaneously. Hence, a geometrical spreading function 1/*R* was adopted, where *R* is the rupture distance. Thus, the following functional form was chosen to analyze the attenuation of PGA, peak ground velocity (PGV), and response spectra with 5 % damping from 0.1 s–5.0 s. Equation (1) shows the function form of attenuation relationship for Ludian earthquake.

1 $$ \lg Y=a- \lg \left({R}_{\mathrm{rup}}+10\right)-b\cdot {R}_{\mathrm{rup}}+\varepsilon $$

**Fig. 9:**
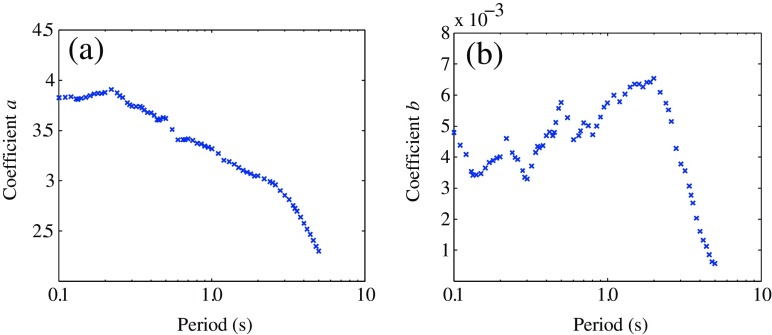
Variation of regression coefficients with natural periods

Where, *a* and *b* are coefficients; *ε* is the residual term following normal distribution with standard deviation of *σ* and mean of 0. The Least squares regression method was used to determine the coefficients and standard deviation of the residuals. The results of regression are shown in Table 4. Figure 9 also shows the variation of regression coefficients with natural periods.

**Table 4:** Coefficients and standard deviations of attenuation model

Parameters	lg*Y* = *a* − lg(*R*rup + 10) − *b*·*R*rup + *ε*		
	*a*	*b*	*σ*
PGA	3.462	0.0042	0.365
PGV	2.425	0.0053	0.346
Sa (*T*) (s)			
0.10	3.827	0.0048	0.368
0.15	3.830	0.0035	0.338
0.20	3.879	0.0040	0.362
0.25	3.847	0.0040	0.380
0.30	3.743	0.0033	0.425
0.35	3.726	0.0043	0.420
0.40	3.674	0.0047	0.433
0.45	3.604	0.0048	0.466
0.50	3.619	0.0058	0.465
0.55	3.510	0.0053	0.462
0.60	3.407	0.0046	0.471
0.65	3.408	0.0047	0.478
0.70	3.416	0.0051	0.483
0.80	3.374	0.0047	0.506
0.90	3.346	0.0053	0.517
1.00	3.318	0.0057	0.497
1.20	3.205	0.0058	0.429
1.50	3.131	0.0064	0.355
2.00	3.050	0.0065	0.297
3.00	2.854	0.0038	0.236
4.00	2.576	0.0016	0.282
5.00	2.301	0.0006	0.284

**Fig. 10:**
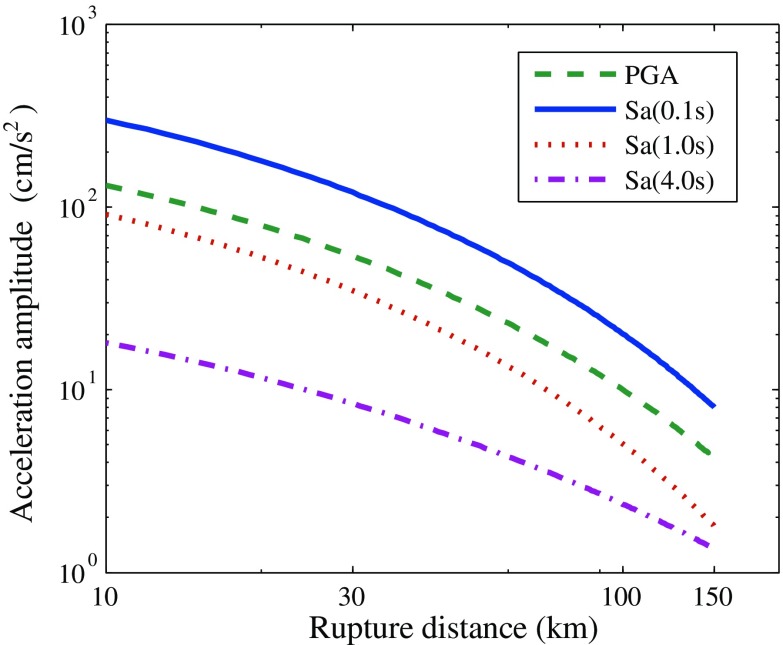
Distance attenuations of ground motion amplitudes for different natural periods

Distance attenuations of PGA and response spectra for some typical periods (*T* = 0.1 s, 1.0 s, and 4.0 s) are compared in Fig. 10. On the whole, the distance decay of short-period ground motion is faster than the long-period ground motions, but the decay rate of acceleration response spectrum for *T* = 1.0 s was significantly larger than the others. It can also be found from Fig. 9b that the coefficient *b* for periods from 0.5 s–2.0 s are markedly greater than those for the other periods.

A regional ground motion prediction equation (GMPE) was developed based on the Wenchuan *M*_w_ 7.9 earthquake and its aftershocks by Zhang et al. (2013). Equation (2) shows the model function of GMPE for western China, and Table 5 shows the model parameters obtained by the random-effect method (Abrahamson and Youngs 1992).

**Table 5:** Model parameters of GMPE for western China

*T* (s)	*α*	*h*	*β*_1_	*β*_2_	*b*	*c*	*M*_c_	*σ*	*τ*
0.0	−0.338	9.015	1.147	−0.242	3.700	−0.317	6.25	0.511	0.279
0.3	0.772	8.232	1.036	−0.142	4.437	−0.382	6.27	0.599	0.278
3.0	1.493	4.130	0.862	−0.199	4.140	−0.815	6.82	0.641	0.462

2 $$ \ln y=f\left(M,{R}_{\mathrm{rup}}\right)+b\cdot SS+{f}_{\mathrm{s}\mathrm{ite}}\left({V_{\mathrm{s}}}_{30}\right)=\alpha \left(M-{M}_{\mathrm{c}}\right)-\left[{\beta}_1+{\beta}_2\left(M-{M}_{\mathrm{c}}\right)\right]\cdot \ln \left(\sqrt{{R_{\mathrm{rup}}}^2+{h}^2}\right)+b\cdot SS+c\cdot \ln \left({V_{\mathrm{s}}}_{30}\right) $$

**Fig. 11:**
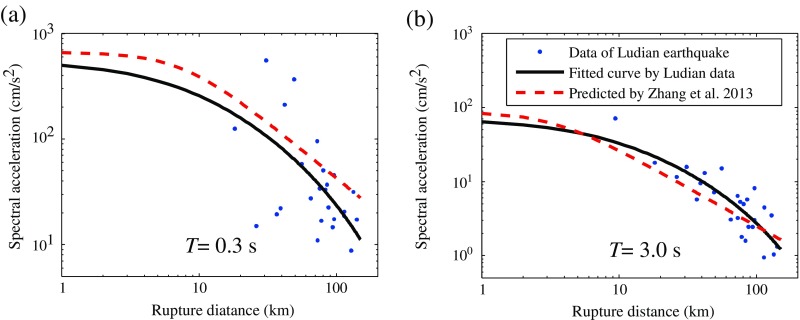
Comparison of attenuation with the GMPE from Wenchuan earthquake

To investigate the attenuation characteristics of the 2014 *M*_s_ 6.5 Ludian earthquake, as well as to observe the GMPE (Zhang et al. 2013) model’s applicability to earthquakes in western China, in Fig. 11, response spectra in short period (*T* = 0.3 s) and long period (*T* = 3.0 s) are compared with the fitted curves using Eq. (1) and the GMPE by Zhang et al. (2013). In the GMPE for western China, the site condition is described by the *V*_s30_ parameter which is a common used parameter in attenuation relationship representing the average shear wave velocity of the top 30 m of the earth. According to the mean value of the selected sites, the *V*_s30_ parameter was set as 360 m/s.

The results indicate that the ground motion amplitudes from the 2014 *M*_s_ 6.5 Ludian earthquake are basically consistent with the predicted results of the GMPE for western China. But at short periods, the amplitudes of Ludian earthquake are slightly smaller than the predicted values. This may result in the strike slip mechanism of the 2014 *M*_s_ 6.5 Ludian earthquake, whereas, the GMPE (Zhang et al. 2013) for western China are developed mainly based on the Wenchuan thrust fault earthquake events.

### Distance dependence of duration

The ground motion duration is also a typical engineering interested parameter; it can reflect the features of the earthquake source, path, and site condition. In this part, the significant duration (*D*_s5–75_) (Trifunac and Brady 1975) defined as time interval between 5 and 75 % of Arias intensity was discussed. The geometric average values of the two horizontal durations are used for analysis.

For a specific earthquake, the distance is the main effect on ground motion duration if the local site effect is not considered. The spreading in time of seismic motion with distance from the source is related to their propagation velocities (Kempton and Stewart 2006). A linear model was adopted to describe the distance dependence of duration according to the previous studies, see Eq. (3) (Esteva and Rosenblueth 1964; Yaghmaei et al. 2014):

3 $$ {D}_{\mathrm{s}5\hbox{-} 75}={c}_0+{c}_1{R}_{\mathrm{rup}}+\eta $$

where *c*_0_ and *c*_1_ are coefficients;*η*is the normal residual with standard deviation *τ*. The results of regression are listed in Table 6.

**Table 6:** Coefficients and standard deviations of duration model

Duration	*D*_s5–75_ = *c*_0_ + *c*_1_ *R*_rup_ + *η*			
	*c*_0_	*c*_1_	*τ*	R^2^-statistic
*D*_s5–75_	4.1537	0.0969	2.3120	0.6759

**Fig. 12:**
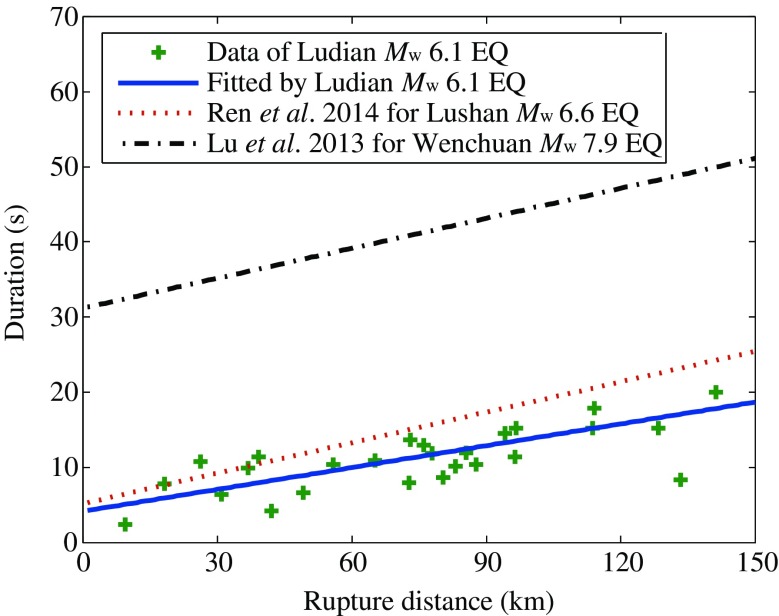
Comparison of distance dependence of duration

As shown in Fig. 12, the duration data (green cross) and fitted curve (blue solid line) of Ludian earthquake are compared with the results of the 2013 Lushan *M*_w_ 6.6 earthquake (Ren et al. 2014) and the 2008 Wenchuan *M*_w_ 7.9 earthquake (Lu et al. 2013). The durations from Wenchuan *M*_w_ 7.9 earthquake are markedly greater than those from Lushan *M*_w_ 6.6 and Ludian *M*_w_ 6.1 earthquakes at all distances. The durations from two Sichuan region earthquakes (Lushan earthquake and Wenchuan earthquake) show similar slope (about 0.135) with distance, which is observably steeper than that for Ludian earthquake (the slope is 0.097) in Yunnan region.

The distance dependence of duration can be interpreted as a result of dispersion of seismic wave. Specifically, seismic waves at different frequencies travel at different velocities and arrive at different time. Therefore, the signals spread out in time domain. With increasing distance, the effect of dispersion enlarges, and the duration increases. Assuming significant duration *D*_s5–75_ is mainly determined by S-wave, so the effect of geometrical dispersion can be neglected. Thus, we can attribute the distance dependence of *D*_s5–75_ to the physical dispersion due to non-elasticity. According to Azimi’s attenuation law (Eq. (4)), the degree of physical dispersion is negatively correlated with quality factor Q.

4 $$ \frac{c\left(\omega \right)}{c\left({\omega}_0\right)}=1+\frac{1}{\pi Q} \ln \left(\frac{\omega }{\omega_0}\right) $$

Where, *c* is phase velocity, *Q* is quality factor of the earth crust.

Incorporating with the companion result of the distance dependence of *D*_s5–75_ from two Sichuan region earthquakes and the 2014 *M*_s_ 6.5 Ludian earthquake in Yunnan region, we can draw a conclusion that Yunnan region has lower physical dispersion and higher *Q* value than that in Sichuan region.

### Site amplification

The local site condition is one of the factors affecting the ground motion. In order to study the effect of soil site on ground motion, we can compare ground motions from soil sites with those from rock sites with the same distance attenuation and source radiation conditions. Unfortunately, it is hard to find a pair of soil site and rock site meeting the aforementioned strict conditions during Ludian earthquake. To address this situation, a method of corrected reference site is used. The attenuation of amplitude with distance is utilized to correct the amplitude of ground motion at rock site so as to remove the effect of geometric attenuation and anelastic attenuation of ground motion. The key point for reference site method is to address the difference of source and path effect on soil site and reference rock site (Choi and Stewart 2005).

According to Borcherdt (2002), for two sites during the same earthquake, if the angle between their connections with the epicenter is less than 30°, the difference of radiation pattern effect on ground motions of the two sites can be ignored. In addition, in near-field region, the anelastic properties of the medium have little effect on the attenuation of ground motion (Abrahamson and Silva 2008), so a geometric attenuation function 1/*R* was used to correct the amplitude of ground motion at reference rock site (Borcherdt 1999). During the 2014 *M*_s_ 6.5 Ludian earthquake, there were some soil and rock sites which met Borcherdt’s requirements. Thus, based on Borcherdt’s method, seven pairs of soil sites and reference rock sites were selected, which are listed in Table 7. Then, site amplification factors can be evaluated by the ratio of amplitudes of ground motions at soil sites and corrected amplitudes of ground motion at the correspondent reference rock sites. The results of soil site amplification factors are also given in Table 7.

**Table 7:** Soil site, reference rock site, and amplification factors in Ludian earthquake

Soil Site	*R*_rup_ (km)	Reference rock site	*R*_rup_ (km)	*T* = 0.1 s	*T* = 0.5 s	*T* = 1.0 s	*T* = 2.0 s
51HDQ	80.3	53DTB	96.4	1.59	1.50	1.82	1.97
53DFZ	133.3	53DTB	96.4	1.61	1.54	1.95	2.15
53HZX	85.5	53DTB	96.4	1.36	1.61	1.71	1.81
53QJX	49.1	53DTB	96.4	1.18	1.67	1.85	2.23
53LLT	9.4	53LDC	18.2	1.58	1.85	1.85	2.19
51HDX	76.0	53QJT	55.8	1.51	1.66	2.16	2.21
53ZJA	39.2	53ZTT	26.2	1.27	2.00	2.18	1.97

**Fig. 13:**
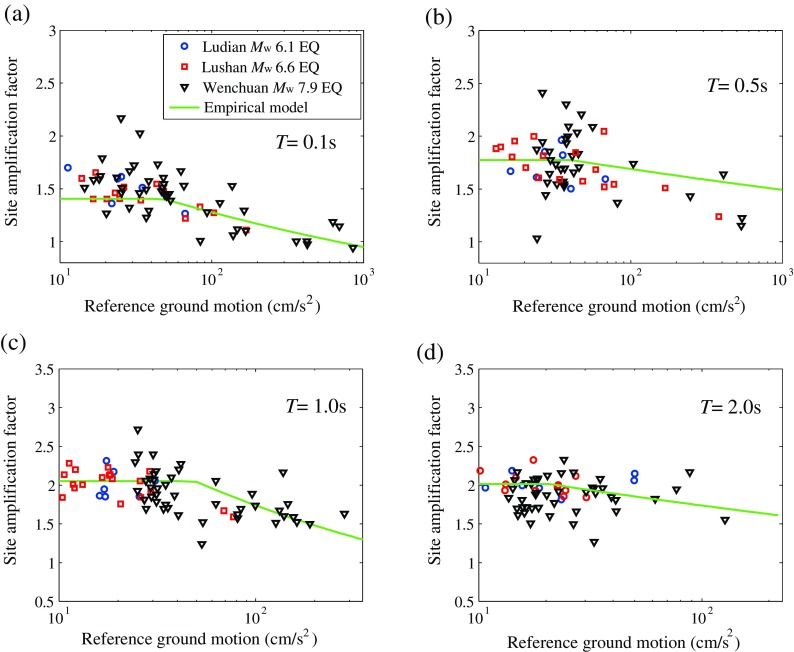
Dependence of site amplification factor on corrected reference rock ground motion

Studies have shown that the ground motion intensity also has significant effect on the site amplification (Walling et al. 2008). Some studies suggested that the site amplification factor and magnitude were significantly correlated (Kamai et al. 2013). But it is also influenced by the soil conditions. Furthermore, amplification effects are more expected to be found for small ground motion stations than for stronger ones, because soil nonlinearity and hysteretic dissipation reduces acceleration peaks. The dependence of site amplification factors on corrected reference rock ground motions from Ludian *M*_w_ 6.1 earthquake, Wenchuan *M*_w_ 7.9 earthquake, and Lushan *M*_w_ 6.6 earthquake are shown in Fig. 13. Where the solid line denotes the empirical site amplification model for western China by Jiang et al. (2013).

For the amplification factors deduced from the 2014 *M*_s_ 6.5 Ludian earthquake, represented by blue circle in Fig. 13, due to the relatively lower magnitude of the earthquake, the reference ground motion is not enough to excite nonlinear behaviors in the soil, so basically, the site amplification factors are in linear stage for Ludian earthquake. Compared with the empirical site amplification model (Jiang et al. 2013), most of the reference ground motion of Ludian earthquake are under the critical value to produce nonlinear amplification in the empirical model. The average amplification factor of Ludian earthquake is consistent with the empirical model.

For the amplification factors deduced from the Wenchuan and Lushan earthquakes, represented by triangle and square respectively in Fig. 13, the site amplification factor decreases with increasing reference ground motion, especially at short periods. The decrease of site amplification with increasing input ground motion can be attributed to the nonlinear seismic response of soil site.

On the one hand, at low reference ground motion level, amplification factors are dominated by linear site response, site amplification is mainly determined by the soil conditions, such as the shear wave velocity and soil thickness. But at high reference ground motion level, the nonlinearity becomes notable with the increasing ground motion amplitude, thus it will diminish the site amplification. On the other hand, for the three earthquakes with different magnitudes, the tendencies of site amplification dependence on reference ground motion are basically similar with the results of empirical model for western China by Jiang et al. (2013).

## Conclusion and discussion

The 2014 *M*_s_ 6.5 (*M*_w_6.1) Ludian earthquake occurred in the eastern Sichuan–Yunnan border region of western China. This earthquake caused serious engineering damage, large economic loss, and high death rate. During the earthquake, the National Strong Motion Network obtained series of acceleration recordings, which provided valuable data to be used in the attenuation relationship studies and characterization of ground motions in western China. Incorporating with the result on the *M*_w_ 7.9 Wenchuan earthquake and the *M*_w_ 6.6 Lushan earthquake, the peak ground acceleration, spectral acceleration, and significant duration parameters were adopted to study the ground motion distribution, attenuation, and site amplification.

The conclusion could be summarized as follows: (1) The ground motion field reveals a directional distribution characteristics along the fault strike, but the asymmetry features of ground motion distribution for hanging wall/foot wall effect is not clear in the 2014 *M*_s_ 6.5 Ludian earthquake; and there appears similar distribution characteristics of the two horizontal components, and the amplitude of vertical component is smaller than the horizontal ones. (2) The ground motion attenuation relationship for Ludian earthquake is basically consistent with the GMPE for western China (Zhang et al. 2013), except that the 2014 *M*_s_ 6.5 Ludian earthquake is slight smaller than the GMPE predicted at short periods, which may attribute to the strike slip mechanism. (3) The distance dependences of duration are similar in Lushan earthquake and Wenchuan earthquake in Sichuan region, but it is different from that of Ludian earthquake in Yunnan region, which may attribute to the lower physical dispersion and higher *Q* value in Yunnan region. (4) The site amplifications for the three earthquakes are basically consistent with the model for western China (Jiang et al. 2013). For lower reference ground motion, the amplification factors are dominated by linear site response, but for high reference ground motion, the nonlinearity becomes notable. The spatial distribution of ground motion, the attenuation characteristics and the site amplification effect should be considered in characterization of near-field ground motion and GMPEs.
